# Using geospatial mapping to predict and compare gambling harm hotspots in urban, rural and coastal areas of a large county in England

**DOI:** 10.1093/pubmed/fdad096

**Published:** 2023-06-30

**Authors:** Mike Saunders, Jim Rogers, Amanda Roberts, Lucy Gavens, Phil Huntley, Sarah Midgley

**Affiliations:** Department of Public Health, Nottingham City Council, Nottingham NG3 3NG, UK; College of Social Science, University of Lincoln, Lincoln LN6 7TS, UK; College of Social Science, University of Lincoln, Lincoln LN6 7TS, UK; Public Health Division, Lincolnshire County Council, County Offices, Lincoln LN1 1YL, UK; Public Health Division, Lincolnshire County Council, County Offices, Lincoln LN1 1YL, UK; Public Health Division, Lincolnshire County Council, County Offices, Lincoln LN1 1YL, UK

**Keywords:** gambling, rural, coastal, urban, maps, geography, information systems

## Abstract

**Background:**

Disordered gambling is a public health problem with interconnections with health and social inequality, and adverse impacts on physical and mental health. Mapping technologies have been used to explore gambling in the UK, though most were based in urban locations.

**Methods:**

We used routine data sources and geospatial mapping software to predict where gambling related harm would be most prevalent within a large English county, host to urban, rural and coastal communities.

**Results:**

Licensed gambling premises were most concentrated in areas of deprivation, and in urban and coastal areas. The aggregate prevalence of disordered gambling associated characteristics was also greatest in these areas.

**Conclusions:**

This mapping study links the number of gambling premises, deprivation, and risk factors for disordered gambling, and highlights that coastal areas see particularly high density of gambling premises. Findings can be applied to target resources to where they are most needed.

## Introduction

There is a growing call to approach gambling from a public health perspective in the UK^1^ as has been undertaken in some other countries.^2^ Gambling is a heavily marketed and commonly participated in activity; the Gambling Commission telephone survey estimates over 40% of people aged at least 16 years in the UK have gambled in the last 4 weeks.^3^ In their 2021 evidence review, Public Health England reported that half of the UK population participates in gambling, with 0.5% of the population experiencing a high level of harm.^4^ Gambling-related harm disproportionately affects disadvantaged and marginalized groups, exacerbates existing health and social inequalities^5^ and intersects with challenges including suicide prevention, alcohol, smoking, interpersonal violence, criminality and homelessness.^6^ Disordered gambling impacts physical and mental health in a range of ways and has significant community and societal costs. Recent estimates in the UK suggest the economic burden of harmful gambling is approximately £1.27 billion, including £342.2 million in mental and physical health harms and £79.5 million in employment and education harms.^7^

Disordered gambling is associated with a greater proximity to and density of in-person gambling facilities,^8^^,^^9^ and a person with disordered gambling is more likely to live in a deprived area,^10^ be unemployed,^11^ smoke, consume alcohol excessively and have mental ill health.^7^ Each person with a gambling disorder has on average 6–10 affected others who may experience relationship strain, stress and financial loss, with interrelated health impacts.^7^

Geospatial mapping is a technique used to display and describe the distribution and variation of information within a specified geography. Modern mapping technologies have been increasingly used to assist in developing public health initiatives,^12^ and visualize social determinants of health in association with rates of health conditions and behaviours, for example in studying the prevalence of non-communicable chronic disease,^13^ and links between alcohol outlet density and violent crime.^14^ Mapping has been used to explore gambling at local levels, including in the UK;^15^^,^^16^ however, previous studies have focused on urban and city locations, and mapping has been at a broad geographical level.

In recent years, local public health teams have shown increasing interest and ambition in gambling harm prevention, yet there are few shared examples of gambling harm mapping being used to inform local public health practice.

Lincolnshire is a large county in England host to urban, rural and coastal communities. Levels of wealth, deprivation and infrastructure vary significantly across the county, and coastal areas experience significant health challenges.^17^ The local prevalence of gambling-related harms was not known. Geospatial mapping techniques were employed as part of a local health needs assessment, to inform and develop a local public health approach to gambling harm.

### Aim

To map gambling-related harm in Lincolnshire using routine data and geospatial mapping to predict ‘hotspots’ of harm, and to compare findings between urban, rural and coastal areas.

## Method

We produced three heat maps of Lincolnshire using QGIS version 3^18^:

1. The location of licenced gambling premises against Index of Multiple Deprivation (IMD) deciles. This is an official measure of relative deprivation in England and is part of a suite of outputs that form the Indices of Deprivation
2. The density of licenced gambling premises
3. The aggregate prevalence of disordered gambling associated characteristics

All data management and analysis were conducted in Microsoft Excel.

### Licenced premises location

We used Gambling Commission data (5 January 2022) to identify premises with a licencing authority named as Boston Borough Council, City of Lincoln Council, East Lindsey District Council, North Kesteven District Council, South Holland District Council, South Kesteven District Council or West Lindsey District Council.^19^ A map pin was placed at the premises’ postcode by importing the postcode data of the premises from Microsoft Excel and assigning a colour corresponding to premises type as reported by the Gambling Commission.^20^ The IMD decile at Lower Super Output Area (LSOA) level was sourced from www.gov.uk and used as background heat mapping.

### Rural, urban and coastal classification

All LSOAs were assigned a classification of ‘rural’, ‘urban’ and ‘coastal’. Rural and urban LSOA were assigned their classification by using the Rural Urban (2011) Classification of LSOA,^21^ produced by Open Geography Portal. Coastal LSOA were assigned their classification by isolating LSOA within areas classified as ‘Coastal Towns’ by the ONS Coastal Towns in England and Wales dataset.^22^ The rate of licenced premises per 1000 population by each classification was calculated using their respective ONS mid-2020 population estimates.^23^

### Density of licenced premises

Premises postcodes were matched to their respective ward or LSOA area. The rate of licenced premises per 1000 population was calculated using their respective ONS mid-2020 population estimates to identify areas where the number of premises per head of population were greatest, which were then visualized onto choropleth maps.

### Disordered gambling associated characteristics

We accessed Public Health England Fingertips data^24^ for variables predictive of disordered gambling in adults, at the lowest geography and most recent year available:

- IMD 2019 (ward)
- Unemployment (percentage of 16–64-year-olds claiming out of work benefit) 2019–20 (ward)
- Percentage prevalence of current smoking in adults (Annual Population Survey) 2019 (district)
- Admission episodes for alcohol-related conditions (Broad—primary and secondary diagnoses): directly standardized rate per 100 000 population 2019/20 (district)
- Estimated percentage prevalence of common mental health disorders (any type of depression or anxiety) in people aged 16 or over 2017 (district)

We hypothesize that areas with a high level of the above factors in combination could indicate greater risk of gambling harm. For each variable, the wards or districts (as relevant to the variable) were ordered from the highest prevalence, rate or IMD score as appropriate to lowest. The areas were ranked according to this order, where the highest prevalence indicated a rank of 1, the next most a rank of 2 and so on. Where data were only available at district level, this was transformed into a ward level rank by assigning a mid-septile value (of 158 wards) to all wards in the corresponding district i.e. wards in the most prevalent district would be assigned Rank 11, wards in the next most are assigned 33 and so on. This avoided district level ranking unfairly influencing the aggregate rank. The rank scores for each ward were summed, and then wards were ordered from the lowest score (highest risk) to highest (lowest risk). The aggregate risk rank score was used to produce a heat map at ward level.

## Results

Lincolnshire is a large county in England with the greatest population count in urban areas, followed by rural and then coastal (Table 1).

**Table 1:** Lincolnshire LSOA classifications (2011)

Classification	Total count of LSOAs	Total population size
Coastal	22	39 935
Rural	190	352 356
Urban	208	374 042
**LINCOLNSHIRE (ALL AREAS)**	**420**	**766 333**

There was an overall county rate of 0.17 premises per 1000 people, with a total of 132 premises (Table 2). Coastal areas had the highest density of gambling premises (1.7 per 1000 population) and highest absolute number of premises, followed by urban areas (0.14 per 1000 population and 54 premises). Rural areas had the lowest density (0.03 per 1000 population).

**Table 2:** Absolute number and rate of premises in coastal, rural and urban areas of Lincolnshire

Classification	Population of area	% Lincolnshire population	Absolute number of premises	Premises per 1000 population
COASTAL	39 935	5.2%	68	1.70
RURAL	352 356	45.9%	10	0.03
URBAN	374 042	48.8%	54	0.14
**LINCOLNSHIRE (ALL AREAS)**	**766 333**	100%	**132**	**0.17**

**Fig. 1:**
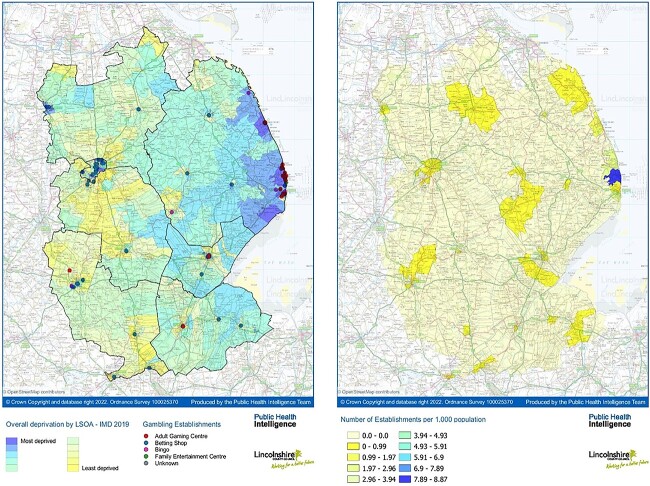
Gambling premises in Lincolnshire: A: licenced gambling premises by type (2021) and IMD at LSOA level (2019); B: number of licenced gambling premises per 1000 population at ward level.

Licenced gambling premises are scattered across the county (Fig. 1). Though not exclusive to areas of deprivation, higher concentrations and clustering of these premises were evident, particularly adult gaming and betting shops, in coastal and urban areas.

There is a higher density of gambling premises in urban (e.g. Lincoln and Grantham) and coastal areas (e.g. Mablethorpe and Skegness). Ingoldmells (coastal) has a notably higher rate of premises per population than other wards.

**Fig. 2:**
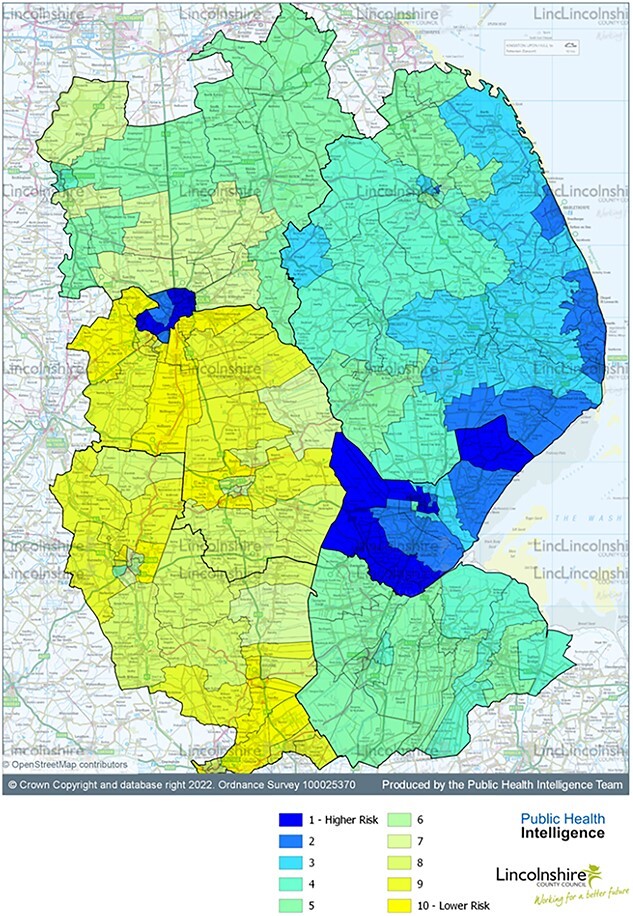
Aggregate prevalence of disordered gambling associated characteristics (IMD, unemployment, adult smokers, alcohol-related hospital admissions, depression or anxiety).

Figure 2 shows the highest aggregate prevalence of disordered gambling associated characteristics in Lincoln, Boston and the east coast. North and South Kesteven generally observe the lowest combined prevalence of risk factors for disordered gambling. When compared with Figure 1A, there is an overlap between the prevalence of disordered gambling characteristics and the location of gambling premises.

## Discussion

### Main findings of this study

The results confirm and enhance findings from earlier studies, which have correlated disordered gambling, deprivation factors and the presence of licenced gambling premises. Coastal areas had the highest number of gambling premises despite being home to only 5.2% of the county population. There was a noticeable difference in rate of premises between area types, with no clear association between the number of premises and population size.

Previous studies had used various mapping techniques to correlate numbers of licenced premises with indices of deprivation, generally in city-based urban areas.^8^^,^^15^ This study adds new knowledge in relation to coastal areas and smaller towns within a more rural area of England. We have found that gambling premises are clustered in coastal and urban areas, and an overlap between the location of gambling premises and characteristics which predict disordered gambling prevalence. These findings can be applied in developing a public health approach to gambling-related harm in Lincolnshire, taking into account differences in access and exposure to in-person gambling premises, and population risk characteristics.

### What is already known on this topic

It is known that there is a higher density of gambling premises in urban areas. Earlier research used mapping techniques to examine gambling premises and disordered gambling-related characteristics in two urban cities.^16^ We have built on this to analyse findings at a county level, including urban, rural and coastal areas. GambleAware has also produced maps to predict areas of gambling-related harm.^15^ Our findings also use more recent data and include small area analyses below Upper Tier Local Authority level, which better translates into intelligence led public health practice.

The mapping for this study also found an unusually high level of premises in coastal areas. There are historical and contemporary explanations, with the popularity of seaside gambling arcades in UK cultural life, and the conversion to gambling of many premises that became available cheaply after the decline of other cultural activities such as theatre going in seaside locations.^25^^,^^26^ The Chief Medical Officer’s 2021 report highlighted coastal population health as one of the most enduring health challenges in the UK^17^, and there is a significant risk this will exacerbate health inequalities.

The UK remains unusual in the global context in the way that we allow children access to gambling opportunities.^27^ Whilst individuals under 18 are generally not allowed to gamble, an exception was made in the Gambling Act 2005 for category D gaming machines. There is a history of widespread availability of and participation in gambling in arcades in seaside towns such as Skegness, by children and families. These arcades contain many of what are now category D machines—these include low-stake fruit machine style machines, coin pushers (sometimes called penny falls) or crane grabs. The machines are found widely in what are known as family entertainment centres, adult gaming centres and pubs, with smaller numbers in other venues such as members clubs, betting shops and casinos.

Emerging evidence clearly connects childhood gambling activities in seaside arcades with disordered gambling as an adult. Newall *et al.*^28^ investigated the links between legal underage gambling (i.e. Category D machines) and disordered gambling symptoms in adults. They questioned over 1000 UK gamblers between the ages of 18 and 40 about their experiences with Category D slot machines, the National Lottery, National Lottery scratchcards, coin push machines and claw grabber machines, all legally available to people under the age of 18. Over 50% of those questioned had interacted with all aforementioned gambling products. Having played a legal gambling product had no association with gambling disorders; however, ‘more frequent use of each of the five products was associated with an increased risk of disordered adult gambling’. Essentially, the more those questioned played with legal forms of gambling under the age of 18, the more likely they were to develop gambling disorders later in life, with claw machines carrying the greatest risk. The frequency of gambling play as children was robustly associated with adult disordered gambling.

### What this study adds

We confirm clear links between numbers of gambling premises, deprivation and certain risk factors for disordered gambling. Whilst unable to directly explore the correlations, they suggest that different place-based factors, including availability of gambling to children in seaside arcades (Ingoldmells) or low levels of education and significant migrant communities in the local population (Boston), may be important to consider in relation to disordered gambling. Some of the coastal, rural and urban issues are significant but not unique to Lincolnshire. This adds to a small but growing literature about what have been called ‘gamblogenic’ environments.^29^

As well as the coastal hotspots, the mapping showed Boston as an area with increased prevalence of characteristics associated with disordered gambling, such as the directly standardized rate of admission episodes for alcohol-related conditions, and the estimated percentage prevalence of common mental health disorders in people aged 16 or over. It should be noted that, as well as the characteristics used for the mapping exercise, Boston is known for having a particularly high proportion of migrants in the population, with the 2021 census showing 23.6% of the current population born outside of the UK (compared to the UK average of 16%).^30^ It is known from studies in a number of countries that migrants may be more vulnerable to gambling harm, and that there is a harm paradox, with lower participation rates, but a disproportionate level of gambling-related harm among those who do participate.^31^

Our findings can be applied in targeting interventions to specific populations at the greatest risk of gambling associated harm, and advocating for integration of gambling harm prevention into work addressing associated risk factors (e.g. mental health or smoking cessation services). The relationship between these factors is however complex and areas of the greatest risk do not necessarily mirror those of the greatest deprivation.^15^

We have highlighted significant issues in coastal and rural areas, and this provides lessons for similar geographies across the country. Our findings may help to inform local licencing policy, and be used to monitor changes to premises density and risk in Lincolnshire over time.

We have found no prior example of local authority public health teams publishing the use of mapping techniques to examine the local distribution and density of gambling premises in comparison with population risk characteristics. We offer a methodology that other public health teams could apply in practice, and use findings to inform and develop a local approach to gambling-related harm.

### Limitations of this study

To produce the findings, a set of proxies that correlate with disordered gambling had to be used in the absence of records of actual disordered gambling at any local level. This highlights a limitation of existing gambling data. Both surveys of prevalence, and data about help seeking, provide some data about demographics but provide only national data, and no available data about the location of those who respond. To properly understand correlations between any place-based factors and disordered gambling, there is an urgent need for better data at local level about the prevalence of disordered gambling and the nature and use of help seeking. The absence of data and knowledge is particularly obvious in relation to rural areas of the UK.^32^

Most gambling-related problems and harm now relate to online gambling.^33^ With 94% of UK adults having access to the internet in 2021, it is not surprising that there has been a gradual switch from people gambling in-person to choosing to gamble online.

GamCare has seen a trend over recent years and now reports more people seeking help for online gambling than for offline gambling. Online gambling increased in 2020/21 due to COVID-19 and has continued post-lockdown. Research by the Gambling Commission shows that participation rates in online gambling have been steadily increasing year-on-year for the past 4 years, with 27% of UK adults gambling online in some form in September 2022, compared with 18.4% in September 2018.^34^ GamCare has seen a trend over a number of years and now reports more people seeking help for online gambling than for offline gambling. In 2021–22, 63% of treatment users were reporting online gambling as their principal form.^30^

It is unknown what impact this is having on rural communities. Individuals living in such communities would have had very limited access to gambling opportunities in the past, and now have gambling accessible all of the time via smartphones and other devices.

We were not able to include online gambling exposure, which would improve understanding of geographical exposure to the products most likely to inflict harm and offer insight to inform targeted work for people experiencing disordered gambling. Routine reporting of remote gambling participation would enhance and facilitate future research.

We have shown important findings for Lincolnshire, and our findings illustrate an issue that needs further study with a larger mapping exercise of other geographies and coastal towns in England.

### Recommendations

This study adds to the growing literature about the importance of considering place-based factors in the development and continuation of behaviours such as disordered gambling. There is a need for more data about the location and residence of gamblers, and those experiencing disordered gambling, in order to better understand which place-based factors relate to deprivation, availability and convenience of different kinds of gambling opportunities, and a range of social and cultural factors.

A significant gap remains in our knowledge of online gambling, and gambling in rural areas. Studies are needed to specifically recruit gamblers in rural areas, and future studies of online gambling should include granular local data about the geographical location of participants.

## Conclusion

Licenced gambling premises were most clustered in urban and coastal areas of a large English county, and with correlation to areas of deprivation and population characteristics predictive of gambling harm. Our study provides data, which may help to target scarce public health resources to where they are most needed, and a methodology for public health teams to apply in developing a local approach to gambling-related harm.

## Data Availability

*The datasets were derived from sources in the public domain:* Gambling Commission. Register of gambling premises [Internet]. 2022. Available from: https://www.gamblingcommission.gov.uk/public-register/premises. Office for Health Improvement and Disparities. Fingertips Public Health Data [Internet]. 2022. Available from: https://fingertips.phe.org.uk/.

## Notes

**M. Saunders,** Specialty Registrar in Public Health

**J. Rogers,** Senior Lecturer in Social Science

**A. Roberts,** Professor of Psychology

**L. Gavens,** Consultant in Public Health

**P. Huntley,** Head of Health Intelligence

**S. Midgley,** Public Health Analyst
